# Methodological issues in qualitative research on HIV prevention: an integrative review

**DOI:** 10.1590/0102-311XEN033123

**Published:** 2023-12-04

**Authors:** Cristiane Spadacio, Lorruan Alves dos Santos, Isa da Silva Sorrentino, Romeu Gomes, Marcelo Eduardo Pfeiffer Castellanos, Eliana Miura Zucchi, Alexandre Grangeiro, Marcia Thereza Couto

**Affiliations:** 1 Faculdade de Medicina, Universidade de São Paulo, São Paulo, Brasil.; 2 Instituto Nacional de Saúde da Mulher, da Criança e do Adolescente Fernandes Figueira, Fundação Oswaldo Cruz, Rio de Janeiro, Brasil.; 3 Hospital Sírio-Libanês, São Paulo, Brasil.; 4 Instituto de Saúde Coletiva, Universidade Federal da Bahia, Salvador, Brasil.; 5 Programa de Pós-graduação em Saúde Coletiva, Universidade Católica de Santos, Santos, Brasil.

**Keywords:** Qualitative Research, Prevention, HIV, Pesquisa Qualitativa, Prevenção, HIV, Investigación Cualitativa, Prevención, VIH

## Abstract

In view of the growing concern about the use of qualitative approach in health research, this article aims to analyze how the qualitative theoretical-methodological framework of HIV prevention is presented in empirical research. We conducted an integrative literature review with the following guiding questions: “How is the qualitative theoretical-methodological framework expressed in empirical research on HIV prevention?”; “What are the limits and potentials of the qualitative methodological designs employed?”. In the qualitative methodological discussion, five dimensions guided the methodological course and the presentation of findings, from the analysis of the characterization of qualitative studies to the contextualization of the studies and the methodological approaches used, highlighting the use of semi-structured interviews with thematic content analysis. We also examined social categories and analytical references, drawing attention to the plurality of these theoretical-conceptual references and to the authors’ polyphony, and identified the limits and potentials of qualitative research. This study focuses on a scientific topic that is related to a wide variety of social groups and analyzes how they are affected by it, examining issues related to social inequality and other analytical possibilities surrounding HIV prevention, and providing resources for a comprehensive methodological discussion. Hence, avoiding the risk of conducting qualitative research based on checklists that limit inventiveness and openness to different designs and forms of execution and analysis is as pivotal as ensuring that the research is consistent and detailed in publications.

## Resumo

Em vista da crescente preocupação com o uso da abordagem qualitativa na pesquisa em saúde, este artigo visa analisar como o referencial teórico-metodológico qualitativo da prevenção do HIV é apresentado na pesquisa empírica. Realizou-se uma revisão integrativa da literatura com as seguintes questões norteadoras: “Como o referencial teórico-metodológico qualitativo se expressa nas pesquisas empíricas de prevenção do HIV?”; “Quais são os limites e o potencial dos desenhos metodológicos qualitativos empregados?”. Cinco dimensões orientaram o fluxo metodológico e a apresentação dos resultados para a discussão metodológica qualitativa, desde a análise da caracterização dos estudos qualitativos até a contextualização destes e as abordagens metodológicas utilizadas, destacando o uso de entrevistas semiestruturadas com análise de conteúdo temático. Além disso, foram examinadas categorias sociais e referências analíticas, destacando-se a pluralidade destas referências teórico-conceituais e a polifonia dos autores, e foram identificados os limites e as possibilidades da pesquisa qualitativa. Assim, esse é um tema científico que articula o estudo com uma diversidade significativa de grupos sociais e como eles são afetados, assim como questões relacionadas à desigualdade social e outras possibilidades analíticas que envolvem a prevenção do HIV, fornecendo recursos para uma discussão metodológica abrangente. Portanto, evitar o risco de realizar pesquisas qualitativas com base em roteiros que limitam a inventividade e a abertura para diferentes desenhos e maneiras de executar e analisar a abordagem qualitativa é tão essencial quanto garantir que a pesquisa seja consistente e detalhada nas publicações.

## Resumen

Ante la creciente preocupación por el uso del enfoque cualitativo en la investigación en salud, este artículo tiene el objetivo de analizar cómo se presenta el marco teórico-metodológico cualitativo de la prevención del VIH en la investigación empírica. Se realizó una revisión integradora de la literatura con las siguientes preguntas orientadoras: “¿Cómo se expresa el marco teórico-metodológico cualitativo en las investigaciones empíricas de prevención del VIH?”; “¿Cuáles son los límites y el potencial de los diseños metodológicos cualitativos empleados?”. Cinco dimensiones han guiado el flujo metodológico y la presentación de los resultados para la discusión metodológica cualitativa, desde el análisis de la caracterización de los estudios cualitativos hasta su contextualización y los enfoques metodológicos utilizados, destacando el uso de entrevistas semiestructuradas con análisis de contenido temático. Además, se examinaron las categorías sociales y los referentes analíticos, destacando la pluralidad de estos referentes teórico-conceptuales y la polifonía de los autores, y se identificaron los límites y las posibilidades de la investigación cualitativa. Así, este es un tema científico que articula el estudio con una diversidad significativa de grupos sociales y cómo son afectados, así como cuestiones relacionadas con la desigualdad social y otras posibilidades analíticas que involucran la prevención del VIH, proporcionando recursos para una discusión metodológica completa. Por lo tanto, evitar el riesgo de realizar investigaciones cualitativas basadas en guiones que limitan la creatividad y la apertura a diferentes diseños y formas de ejecutar y analizar el enfoque cualitativo es tan esencial como asegurar una investigación consistente y minuciosa en las publicaciones.

## Introduction

Qualitative research has its theoretical-methodological foundations established on references from the Social and Human Sciences, displaying analytical concerns about the meanings attributed to subjects’ experiences in their social interaction contexts ^1^. It seeks to apprehend the representations, beliefs, perceptions, opinions, and other factors resulting from the interpretations and experiences of subjects in their social-historical contexts.

Epidemiological or clinical studies usually cannot fully support health prevention, especially HIV prevention. Knowing the profile of the population at risk of HIV infection, of people living with HIV and the clinical picture associated with HIV may not be good prevention strategies. The effectiveness of HIV prevention policies, programs, and actions also depends on evidence from qualitative studies guided by social theories ^2^.

Although these types of studies are already incorporated ^3^ ^,^ ^4^, their use in the formulation, implementation, and guidance of prevention policies is still incipient. They occupy a complementary or secondary place in analysis frameworks shaped by evidence from epidemiological and clinical studies, which strongly emphasize biomedical knowledge ^5^. Frequently, meta-syntheses in the field of HIV prevention focus on subjects’ knowledge, access, and experiences related to the use of preventive methods or social and legal dimensions that cross prevention policies ^6^.

Especially in Public Health, there are concerns about the quality of qualitative research: its conception, methodological development, and outcomes disclosure are questioned both by researchers and by scientific journals ^7^ ^,^ ^8^. Despite different editorial orientations, these show the necessary explanation of the main aspects that must be reported in publications of qualitative studies. Although no consensus have been reached on the definition of quality criteria for qualitative research ^8^, challenges constantly arise around this discussion ^9^.

Taking into account these methodological concerns surrounding qualitative research, this study is based on the following guiding questions: “How is the qualitative theoretical-methodological framework expressed in empirical research on HIV prevention?”; “What are the limits and potentials of the qualitative methodological designs?”. In this sense, the general objective of this article is to analyze how the qualitative theoretical-methodological framework presents itself in empirical research on HIV prevention, pointing out limits and potentials for future research in this field.

## Methods

**Figure 1:**
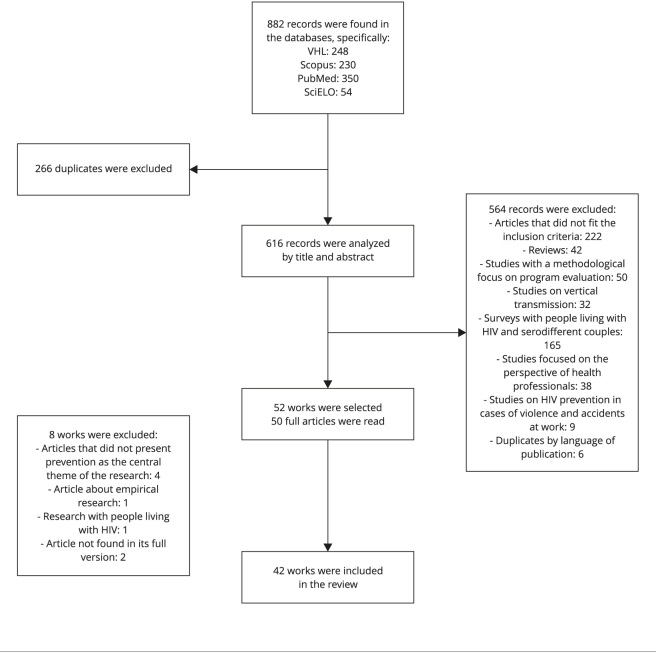
Selection flowchart of articles included in the literature review.

We performed an integrative review of the literature on HIV prevention via published empirical articles ^10^ with a qualitative methodological approach, and applying the ENTREQ Protocol ^11^ (Figure 1).

VHL: Virtual Health Library.

We selected references from the Virtual Health Library (VHL), SciELO, PubMed, and Scopus databases, basing choices on syntax and using descriptors previously validated in DeCS (Health Science Descriptors)/MESH (Medical Subject Headings): (HIV) AND (prevention) AND (qualitative research) (Table 1). The decision to use validated descriptors instead of search terms, which was adopted by some indexed journals, resulted in the exclusion of studies that could have been included in our review but were not captured in the selection based on descriptors. The advanced search considered the field titles and abstracts in the previous selection without time restriction. We were unable to access two articles, even after contacting the main authors. The search process was conducted from July to August 2022.

**Table 1:** Search matrix.

Database	Search strategy used	Records found [n (%)]
SciELO	(HIV) AND (prevention) AND (“qualitative research”)	54 (6.1)
PubMed	((hiv[Title/Abstract]) AND (prevention[Title/Abstract])) AND (“qualitative research”[Title/Abstract])	350 (39.7)
VHL	(hiv) AND (prevention) AND (“qualitative research”) AND ( type_of_study:(“qualitative_research”))	248 (28.1)
Scopus	(hiv) AND (prevention) AND (“qualitative research”)	230 (26.1)

VHL: Virtual Health Library.

Original articles considered for inclusion presented results of empirical qualitative research on HIV prevention, focusing on the perspective of population groups. Literature review studies were excluded, as well as studies methodologically evaluating programs and public policies focused on the perspective of health professionals or other prevention agents, studies on perinatal transmission of HIV, surveys with people living with HIV and serodifferent couples , and studies related to the exposure to HIV through sexual violence or occupational accidents. The Rayyan online platform (https://www.rayyan.ai/) was used to the selection of articles.

After our final selection, we included 42 articles in this review and created five dimensions to extract their data and analyze the potentials and limits of qualitative research in the health care field:

(1) Characterization of publications (year of publication, journal, corresponding author’s institution/country and gender);

(2) Contextualization of qualitative studies (objectives, referenced theoretical framework, definition of the study object, study groups/populations);

(3) Methodological approaches used (study design, methods or techniques, justification for data sources, sample description, data analysis, program used for data analysis, ethical issues);

(4) Social categories and references used for analysis (theoretical approach to methodology and social categories as references for analysis, social categories and references);

(5) Limits and potentials identified in the qualitative research.

## Results

Analyzing the first established dimension, we found that the publication date of the chosen studies ranged from 2011 to 2020, and that the studies were conducted in various regions and countries, such as the United States (n = 14) and Brazil (n = 9). The articles were published in different journals from disciplinary and thematic areas of public health. Based on the names of the leading authors, we found that women were the preeminent authors in most publications (26 out of 42).

While examining the second dimension of the analysis (contextualization of qualitative studies), we noticed that all articles presented their research objectives. Although 15 of the 42 papers did not clarify which theoretical framework was chosen for the research design, almost all articles included references to previous studies in the field of HIV prevention. Articles explaining the chosen theoretical framework relied on references such as theoretical-conceptual frameworks of hegemonic masculinity ^12^ ^,^ ^13^; risk ^14^; vulnerability ^15^; the theory of social representations ^16^ ^,^ ^17^ ^,^ ^18^; medical anthropology ^19^ ^,^ ^20^; a theoretical framework of the intergenerational dialogues model ^21^; African womanism and the Afrocentric theory ^22^; the socioecological model ^23^ ^,^ ^24^; studies anchored in the Health Belief model; and the information-motivation-behavioral skills model ^25^ ^,^ ^26^.

Regarding definition of the object, the dimensions investigated were varied. Some studies focused on constructs or mediating processes related to the use of preventive methods and/or to the participation in preventive interventions. When this was the case, information, knowledge, social representations, beliefs, attitudes, decision-making, perceptions, and acceptability constituted investigation objects. Other studies investigated care experiences related to preventive methods, including the use and adherence to such methods and patients’ interactions with health professionals and services ^12^ ^,^ ^13^ ^,^ ^24^ ^,^ ^25^ ^,^ ^26^ ^,^ ^27^ ^,^ ^28^ ^,^ ^29^ ^,^ ^30^ ^,^ ^31^ ^,^ ^32^ ^,^ ^33^ ^,^ ^34^ ^,^ ^35^. We investigated subjects’ experiences with these preventive methods and practices at the interface with different social groups, such as heterosexual men ^13^ ^,^ ^36^ ^,^ ^37^ ^,^ ^38^; men who have sex with men (MSM) ^25^ ^,^ ^30^ ^,^ ^39^ ^,^ ^40^ ^,^ ^41^ ^,^ ^42^ ^,^ ^43^; women ^26^ ^,^ ^33^ ^,^ ^44^ ^,^ ^45^ ^,^ ^46^; African-Americans ^21^; women over 50 years ^19^ ^,^ ^24^; young people (cisgender, transgender, MSM) ^16^ ^,^ ^27^ ^,^ ^34^ ^,^ ^35^ ^,^ ^40^ ^,^ ^47^ ^,^ ^48^; and sex workers ^23^ ^,^ ^29^ ^,^ ^45^. Still at the interface with preventive measures, some studies have analyzed issues related to vulnerability ^15^ ^,^ ^22^ ^,^ ^49^; explained risk behavior as the central object for analysis ^14^ ^,^ ^31^ ^,^ ^35^ ^,^ ^39^ ^,^ ^42^ ^,^ ^47^ ^,^ ^50^ ^,^ ^51^ ^,^ ^52^; masculinity ^12^ ^,^ ^27^ ^,^ ^28^ ^,^ ^36^; gender ^22^ ^,^ ^50^ and sexuality ^16^ ^,^ ^43^ ^,^ ^53^ ^,^ ^54^; and social support networks ^41^.

Our third dimension of analysis investigated different methodological approaches. Among the articles we selected, some arose from research conceived as exclusively qualitative ^12^ ^,^ ^19^ ^,^ ^21^ ^,^ ^22^ ^,^ ^25^ ^,^ ^26^ ^,^ ^33^ ^,^ ^34^ ^,^ ^35^ ^,^ ^36^ ^,^ ^37^ ^,^ ^38^ ^,^ ^43^ ^,^ ^45^ ^,^ ^46^ ^,^ ^47^ ^,^ ^48^ ^,^ ^53^ ^,^ ^54^; some were based on articles with a mixed design ^15^ ^,^ ^16^ ^,^ ^27^ ^,^ ^32^ ^,^ ^41^ ^,^ ^53^; and some reported having a “qualitative” focus on the “phase” or “component” of broader research ^13^ ^,^ ^20^ ^,^ ^24^ ^,^ ^28^ ^,^ ^29^ ^,^ ^30^ ^,^ ^31^ ^,^ ^39^ ^,^ ^40^ ^,^ ^44^ ^,^ ^47^ ^,^ ^52^, such as clinical trials and community-based research.

As for the explanation of the theoretical anchoring of the different methodological designs, we found studies that were based on ethnography ^20^ ^,^ ^39^ ^,^ ^54^; medical anthropology ^19^; interpretive anthropology ^15^; case study ^47^; phenomenological study ^43^; community-based study ^34^; and the Theory of Social Representations ^16^. However, many studies lacked an explanation of the methodological design, citing only the methodological approach and the techniques for production and analysis ^12^ ^,^ ^13^ ^,^ ^14^ ^,^ ^21^ ^,^ ^22^ ^,^ ^24^ ^,^ ^25^ ^,^ ^26^ ^,^ ^27^ ^,^ ^28^ ^,^ ^29^ ^,^ ^30^ ^,^ ^31^ ^,^ ^35^ ^,^ ^37^ ^,^ ^38^ ^,^ ^40^ ^,^ ^42^ ^,^ ^43^ ^,^ ^44^ ^,^ ^45^ ^,^ ^46^ ^,^ ^48^ ^,^ ^49^ ^,^ ^50^ ^,^ ^52^ ^,^ ^53^.

As for the production of empirical data, the most used data production techniques in the research were in-depth interviews with a semi-structured guide ^12^ ^,^ ^13^ ^,^ ^14^ ^,^ ^15^ ^,^ ^21^ ^,^ ^24^ ^,^ ^25^ ^,^ ^26^ ^,^ ^27^ ^,^ ^29^ ^,^ ^31^ ^,^ ^32^ ^,^ ^33^ ^,^ ^36^ ^,^ ^37^ ^,^ ^40^ ^,^ ^43^ ^,^ ^44^ ^,^ ^46^ ^,^ ^48^ ^,^ ^51^ and focus groups ^30^ ^,^ ^34^ ^,^ ^35^ ^,^ ^50^ ^,^ ^53^, often combined in studies ^23^ ^,^ ^28^ ^,^ ^38^ ^,^ ^41^ ^,^ ^43^ ^,^ ^45^ ^,^ ^47^ ^,^ ^52^ or narrative interviews ^12^ ^,^ ^19^. Anthropologically-oriented studies used field diaries as a tool for collecting information, along with other strategies, such as semi-structured interviews ^19^ ^,^ ^22^ ^,^ ^39^ ^,^ ^54^. In studies with online surveys, data collection technologies were also employed ^16^ ^,^ ^32^ ^,^ ^35^ ^,^ ^53^.

Qualitative research is expected to align data collection and analysis techniques with specific theoretical references that guide analytical procedures; however, eight out of the 42 selected studies did not report which references guided the analyses. In the studies that did report these references, different approaches were proposed, such as the Grounded Theory ^25^ ^,^ ^31^ ^,^ ^40^ ^,^ ^42^ ^,^ ^47^ ^,^ ^50^ ^,^ ^51^, the Ecological Model Theory ^24^ ^,^ ^47^, and content analysis ^21^ ^,^ ^41^ ^,^ ^53^ ^,^ ^54^. Thematic analysis was also referenced among the studies ^26^ ^,^ ^30^ ^,^ ^34^ ^,^ ^37^ ^,^ ^45^. Some studies were also founded on the method of interpretation of the senses, based on hermeneutic-dialectical principles ^12^ ^,^ ^13^, on the Theory of Social Representations ^16^ ^,^ ^46^, and on phenomenology ^27^.

In almost all studies, thematic analyses of the corpus of transcribed contents were performed, with the codification and construction of analytical categories. As support for data analysis, 29 of the 42 studies used one of the following software: QSR Nvivo (in its different versions); Ensemble de Programmes Permettant L’Analyse des Evoctions (EVOC); Atlas.Ti; ALCESTE, HYPERBASE; Dedoose, Ethnograph 5.0; Tri-Deux Mots version 2.2; and QSR-N6.

The ethical aspects involved in the research development were reported in 29 of the 42 studies. In some of them, only the approval by research ethics bodies/committees was mentioned (16), while in others, the adopted ethical procedures were described in detail (13).

Through our fourth established dimension, which included the social categories and references for analyses in the studies, we sought to map, among other aspects, the segments and classifications of social differentiation included in the articles. A diversity of social categories was also included in the theoretical-methodological models that guided the analyses. Some journals explicitly mentioned their chosen social categories while describing their methodological concerns ^12^ ^,^ ^13^ ^,^ ^14^ ^,^ ^16^ ^,^ ^19^ ^,^ ^21^ ^,^ ^22^ ^,^ ^28^ ^,^ ^32^ ^,^ ^34^ ^,^ ^38^ ^,^ ^40^ ^,^ ^41^ ^,^ ^42^ ^,^ ^47^ ^,^ ^49^ ^,^ ^50^ ^,^ ^51^. Among these categories, gender stood out in various studies, such as those dealing with hegemonic masculinity ^12^ ^,^ ^13^ and African-American women ^22^. Gender was also intertwined with other social markers ^13^ ^,^ ^33^; such as socioeconomic statuses ^34^. We also identified the sexuality category, present in discussions about the impact of racism and socioeconomic status on sexual behavior ^15^ ^,^ ^50^. Some articles discussed the exposure of populations to social vulnerabilities ^12^ ^,^ ^14^ ^,^ ^15^ ^,^ ^40^ ^,^ ^47^ ^,^ ^49^ ^,^ ^50^ and sought to link different social categories in their analyses: an example is a study that identified how people of different ethnic-racial identities, genders, and ages can be more or less vulnerable to HIV.

During the analysis of our last dimension (limits and potentialities of the researches), we found that some articles did not mention the impact(s) of their methodology on the research results ^14^ ^,^ ^16^ ^,^ ^28^ and suggested new studies or interventions on the initial themes ^12^ ^,^ ^19^ ^,^ ^21^ ^,^ ^27^ ^,^ ^29^ ^,^ ^39^ ^,^ ^41^ ^,^ ^42^ ^,^ ^48^ ^,^ ^50^ ^,^ ^53^ ^,^ ^54^. Other articles indicated the importance of studies for drawing up public policies focused on HIV prevention ^13^ ^,^ ^15^ ^,^ ^23^ ^,^ ^30^ ^,^ ^35^ ^,^ ^37^ ^,^ ^45^ ^,^ ^49^, and some reported that the findings evidenced through the qualitative methodology were essential for the development of interventions, serving as a guidance for public polices and prevention programs ^36^ ^,^ ^44^ ^,^ ^46^ ^,^ ^47^ ^,^ ^52^. Some studies discussed the limitations of the findings of qualitative research: one of them raised concerns over the possible generalization of results of prevention strategies for young MSM ^40^, and another signaled the low number of participants in researches on injectable drug users’ experiences with HIV prevention ^51^.

## Discussion

Our integrative review included 42 articles selected from the largest and most expressive health databases in the world. Most of the articles (19) were published between 2011 and 2020, and publications were made both in public health journals and in journals specialized in HIV prevention.

The majority of the analyzed studies were conducted in the United States and in Brazil, and had mostly female authors.

We hypothesize that women prevailed in the authorship of the selected articles because the women’s movement, along with the activism related to AIDS and with the gay movement, has played an important role in actions aimed at policies, interventions, and prevention actions focused on HIV. In this sense, the women who dealt with this issue, regardless of their sexual orientation, joined other movements, becoming active both in the demanding groups’ demonstrations and in the academic research on the subject. Another factor that may have contributed to this female prevalence is the geographical area in which the studies were conducted: in Brazil, for example, women are the majority (60%) of researchers in Life and Health Sciences, while in Computer Sciences and Mathematics the female presence does not even reach 25%. In terms of scientific production in general, from 2014 to 2017, Brazil was the Ibero-American country with the most women signing scientific articles - females represented 72% of the total authors ^55^ ^,^ ^56^.

Publications of selected qualitative studies on HIV prevention began to appear in the late 1990s, increasing in the first decade of the 2000s and growing even more expressively during the year 2021. Many studies that originated these publications were conducted before the COVID-19 pandemic, including longitudinal studies and some with mixed methodologies. However, in the first six months of 2022, publications began to report studies made during the pandemic, usually conducted remotely and using information technologies. This last increase probably occurred due to a worsening concern over the access to services for sustaining HIV prevention strategies in the context of the pandemic ^57^. Even so, the increase in publications about HIV from the 2000s onwards demonstrates that studies on the subject have been intensified, possibly in response to new global trends in the epidemic and to the use of new technologies and institutional programs aimed at HIV prevention.

Furthermore, we observed that the profile of participants varied widely across studies, both in the categories of social differentiation included in the theoretical-methodological models and in the data analyses. Different theoretical frameworks were also employed to support the conducted analyses. However, most studies significantly lack details describing the theoretical framework used for their research design.

The diverse social groups included in the studies portrays the populations that are the focus of most HIV prevention initiatives, and sections of highly vulnerable groups (young transgender people, young refugees, people who are homeless, and MSM in rural areas). Qualitative research is highly important, as it allows for advances in the general understanding of experiences and vulnerability contexts linked to HIV, which contributes to the development of prevention policies, especially those aimed at groups undergoing a systematic violation of human rights and those addressing the resurgence or stagnation of the HIV epidemic. At the same time, there is a noteworthy absence of research on the use of ARV-based HIV prevention methods by young people and adolescents, and of research with trans men, a social group that is often not reached by prevention policies.

Most studies we analyzed were exclusively qualitative. The second most used methodological approach was that of mixed methods, including a qualitative component, of explanatory and exploratory models, and the third was the use of a qualitative component as part of broader research with different methods. We also observed a predominance of analyses of participants’ experiences with prevention measures, and interviews and focus groups stood out as frequently used data production techniques. Lastly, most studies did not provide clear descriptions of the criteria used for determining sample size, and limited the description of ethical aspects to a mere citation of the protocol approval number. In this way, a very traditional approach seems to predominate in data production and in the engagement of more vulnerable populations in the research process.

Research with people in vulnerable contexts requires less conventional methodologies and approaches - which is not only a matter of methodology, but also one of ethics. The use of methods that are not appropriate or sufficiently adapted to consider social vulnerabilities can result in the exclusion of vulnerable populations from research. Those who are unwilling to go beyond traditional methodological limits perpetuate a research culture in which vulnerable groups and people are neglected, considered too “difficult” to be included in research. In this sense, methodological diversification is a tool for promoting more empathetic and democratic research participation, and includes the use of approaches that provide greater visibility and stimulates participants’ resilience instead of their susceptibility ^58^.

During the methodological analysis, we also found that the selected studies displayed a plurality of theoretical-conceptual references, which could indicate a profusion of perspectives from different study fields (cultural, gender, decolonial studies, Social Psychology, Medical Anthropology, Public Health) and a polyphony of authors.

In most of the selected studies, the authors conducted analyses of the empirical material from the corpus of transcribed content, using different thematic analysis strategies and frequently employing information resources (software) to support such analyses. Some authors particularly linked their analyses to further contextual analyses of the investigated social categories. The term “social categories” is traditional in the fields of Social Sciences and Humanities in Health, and even after four decades of studies in this area on HIV and prevention, recent articles continue to use the term “social markers” without major concerns regarding how it is considered in filiation theory and in more recent studies, such as those on intersectionality. The (inter)relations between markers such as social class, gender, race/ethnicity, sexual orientation, among others, must be analyzed in historical conjunctures and specific social situations, and authors should keep in mind the idea that differences crosscut social actions, both to determine how much to enable changes in the meaning of these actions promoted by cultural practices of resistance and recreation of the social world ^59^.

## Final considerations

This article results from the scientific community’s growing concern over the characteristics of qualitative research. We assessed the quality of different qualitative researches published in scientific journals on health and the lack of determination in their design, conduction, and analysis. Scientific journals have incorporated the use of checklists to provide greater integrity and transparency in the report of qualitative research, but this practice has been the subject of intense discussion ^7^. It is necessary to ensure that research is reported in a coherent and detailed manner, so that it is possible to evaluate it according to the value of the research product, that is, based on criteria of originality, substantiveness, and contributions that are not foreseen in checklists, not least because they are not - and cannot be - their purpose. Avoiding the risk of guiding qualitative research on checklists that restrict inventiveness and openness to different designs is as important as ensuring that the research is performed coherently and reported in detail in publications, and also allows for the emergence of various ways of conducting and analyzing the context of the qualitative approach.

HIV prevention studies based on qualitative approaches have high scientific relevance, as they articulate a relevant diversity of social groups, revealing their struggles by exposing issues related to social inequality, social justice, and human rights, recurrent and important aspects surrounding HIV prevention.

This study has both strengths and limitations. As strengths, we can highlight the search strategy, which was designed to be sensitive to all qualitative publications in the area and did not have any time or language restrictions, thus further reducing the chances of excluding publications that met the adopted eligibility criteria. The use of descriptors limits access to articles and journals that use keywords. In addition, we conducted the search in the largest and most representative health databases. The objective of our analysis was to examine methodological aspects of qualitative articles on HIV prevention, but it is impossible to summarize the results obtained in the researches conducted by all the selected studies. We encourage the creation of future literature reviews with the same objective as ours. Given the growing interest in high-quality qualitative research in health studies, our contribution goes beyond the specific contributions of this type of approach in the field of HIV prevention. Given the volume and complexity of studies on HIV prevention, which have the potential to effectively address the HIV pandemic, it is necessary to reflect on the importance of qualitative research and of its contributions to the development of this relevant field of knowledge and practice.
